# Intraoperative optical coherence tomography-guided nanothin Descemet stripping automated endothelial keratoplasty in a patient with a remarkably thickened cornea

**DOI:** 10.1016/j.ajoc.2022.101414

**Published:** 2022-02-10

**Authors:** Hideaki Yokogawa, Akira Kobayashi, Natsuko Mori, Tsubasa Nishino, Haguku Nozaki, Kazuhisa Sugiyama

**Affiliations:** a Department of Ophthalmology, Kanazawa University Graduate School of Medical Science, Kanazawa, Japan; b Department of Ophthalmology, Saiseikai Kanazawa Hospital, Kanazawa, Japan; c Department of Ophthalmology, Toyama Prefectural Central Hospital, Toyama, Japan

**Keywords:** Descemet-stripping automated endothelial keratoplasty, Nanothin, Intraoperative optical coherence tomography

## Abstract

**Purpose:**

To report use of intraoperative optical coherence tomography (OCT) for nanothin Descemet stripping automated endothelial keratoplasty (DSAEK) in a patient with an extremely thickened cornea due to advanced bullous keratopathy.

**Observations:**

A 90-year-old woman with a history of multiple trabeculectomies was referred to us for treatment of advanced bullous keratopathy (1400 μm central corneal thickness). Nanothin DSAEK was planned and performed. In brief, after the removal of the loose corneal epithelium, the anterior chamber was meticulously observed using a surgical microscope and oblique light via an endoillumination probe; however, the visibility of the anterior chamber was limited because of severe corneal edema. Subsequently, a nanothin (47 μm) DSAEK graft stained with trypan blue was inserted into the anterior chamber using an NS endoinserter. Intraoperative OCT was used successfully to visualize the graft unfolding, air tamponade, and graft attachment. At 3 months postoperatively, significant corneal clearing (625 μm central corneal thickness), improvement of visual acuity (decimal 0.04), and pain relief were obtained.

**Conclusions and importance:**

Intraoperative OCT is useful for nanothin DSAEK even when the surgical microscope view is compromised by a remarkably thickened host cornea due to advanced bullous keratopathy. As an alternative to a penetrating keratoplasty, less invasive nanothin DSAEK was successfully performed.

## 1 Introduction

In recent decades, endothelial keratoplasty, rather than conventional penetrating keratoplasty (PK), has been the initial choice for surgical treatment of corneal endothelial diseases.^1^^,^^2^ Advantages of endothelial keratoplasty over PK include faster visual recovery and lower risk of traumatic globe rupture, suture complications, and allograft rejection.^1^, ^2^, ^3^, ^4^, ^5^ Currently, the two types of major endothelial keratoplasty are Descemet stripping automated endothelial keratoplasty (DSAEK) and Descemet membrane endothelial keratoplasty (DMEK). Nanothin DSAEK was introduced to use a ≤50 μm graft that enables a minimal posterior stromal tissue transplant.^6^^,^^7^

Intraoperative optical coherence tomography (OCT) has been employed for lamellar corneal surgery^8^, ^9^, ^10^, ^11^ and other types of ocular surgeries. Microscope-integrated OCT can visualize real-time B scan images, and facilitate surgical decision-making during the operation. The current study reports the use of intraoperative OCT to complete DSAEK using a nanothin graft in a patient with a remarkably thickened cornea due to advanced bullous keratopathy.

### 1.1 Case report

**Fig. 1:**
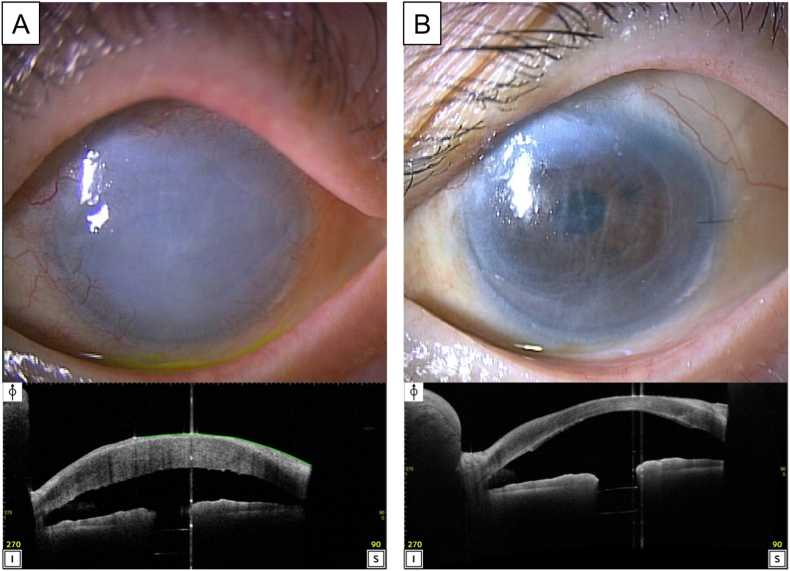
Pre- and postoperative images of the left eye of a 90-year-old woman. **(A)** Preoperatively, this eye had a remarkably thickened cornea due to advanced bullous keratopathy. Although it was difficult to evaluate the anterior chamber with a slit lamp biomicroscope, an anterior segment OCT image shows the open angle, adequately small pupil, and well-fixed posterior chamber intraocular lens. The central corneal thickness was 1400 μm. **(B)** At 3 months after nanothin DSAEK, significant corneal clearing was obtained. Host Descemet membrane striae persisted at the host–graft interface. The central corneal thickness was reduced to 625 μm. There were no remarkable changes in slit lamp findings through at least 18 months of follow-up.

**Fig. 2:**
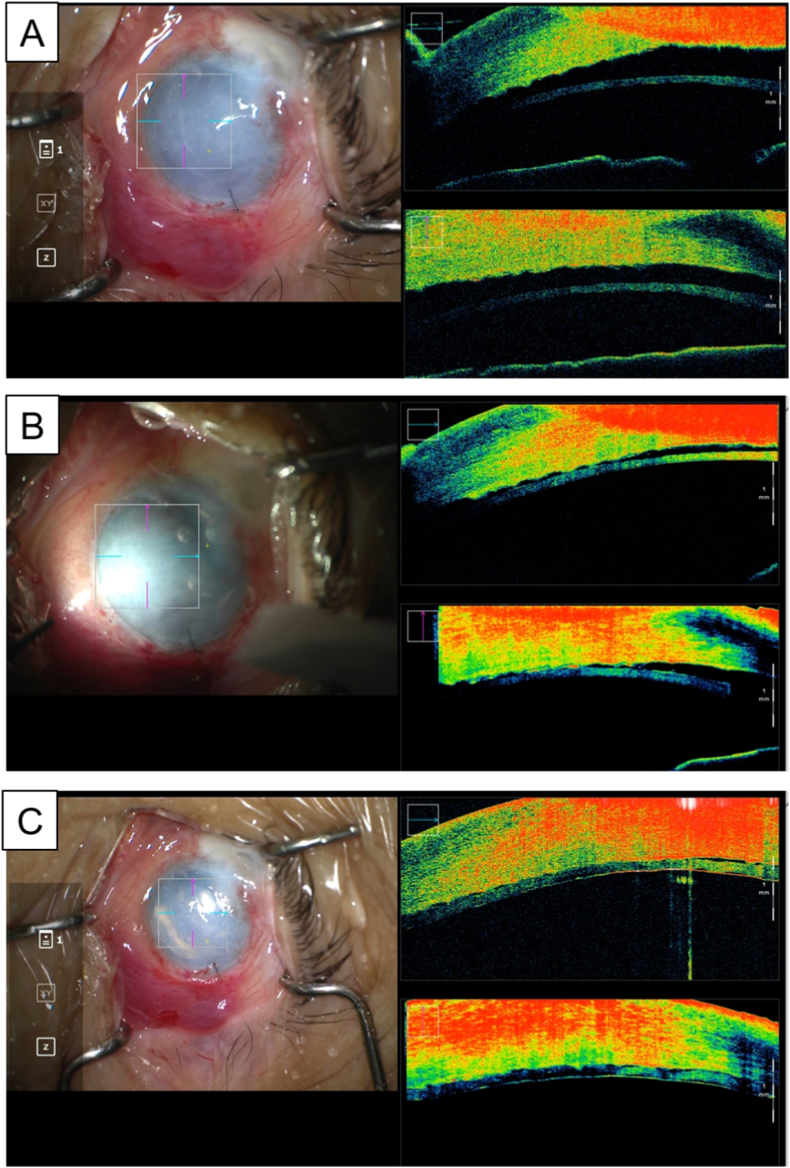
Intraoperative images of nanothin DSAEK using a microscope-integrated OCT. **(A)** After the trypan blue-stained nanothin graft insertion, the inserted graft was not visible with a standard microscope view on the left because of severe corneal edema, whereas the inserted graft was visible with an OCT B-scan view on the right. **(B)** The inserted graft was faintly visible under oblique light via an endoillumination probe with a standard microscope view on the left. **(C)** At the end of the surgery, the air reflection was visible in a standard microscope view on the left. Correct positioning of the nanothin DSAEK graft is confirmed in an OCT B-scan view on the right. (For interpretation of the references to colour in this figure legend, the reader is referred to the Web version of this article.)

A 90-year-old woman was referred to us for treatment of advanced bullous keratopathy (1400 μm central corneal thickness) in her left eye (Fig. 1A). She had late-stage glaucoma with multiple trabeculectomies. Her best corrected visual acuity (BCVA) was decimal 0.8 OD and hand motion OS, and her intraocular pressure was 15 mmHg OD with antiglaucoma eye drops and 10 mmHg OS without. Preoperative anterior segment OCT image showed no significant irregularities of the posterior cornea or iris-lens abnormalities. After obtaining informed consent for less invasive DSAEK rather than PK, we performed a nanothin DSAEK procedure for her left eye (Fig. 2). At the beginning of surgery, the loose epithelium was removed for better visualization, and a temporal corneal incision 4.6 mm wide was created. Neither host descemetorhexis nor iridectomy was performed. A nanothin donor graft, which had been cut at 47 μm thickness using a single pass microkeratome at the US Eyebank (Corneagen, Seattle, USA) was prepared using a 7.0 mm diameter punch, and stained with trypan blue for visualization. The graft was not marked with S or F stamp.^12^ The graft was loaded and inserted into the anterior chamber using an NS endoinserter (Hoya, Japan). Intraoperative OCT (Rescan 700, Carl Zeiss Meditec) was used to visualize the graft unfolding in the anterior chamber. Under endoillumination, a 27 gauge blunt-needle was inserted into the anterior chamber, and a small amount of air was gently injected to press the graft against the posterior surface of the host cornea. After adequate air injection, intraoperative OCT was used to confirm the correct position of the graft. The shape of the graft edge on OCT demonstrated stromal-side up (acute-angled bevel sign).^13^ A vent incision to remove the interface fluid was not made. At the end of the surgery, steroid (1.65 mg dexamethasone) was injected under the conjunctiva and 0.3% ofloxacin and 0.1% betamethasone ointment were applied. The patient was instructed to keep supine for at least 1 hour.

Postoperatively, topical 0.5% levofloxacin and topical 0.1% betamethasone were applied 5 times a day, and then the eye drops were tapered. At 3 months postoperatively, significant corneal clearing with 625 μm central corneal thickness, improvement in BCVA (decimal 0.04 for late-stage glaucoma), and pain relief were obtained (Fig. 1B). Intraocular pressure was 7 mmHg. Endothelial cell density (ECD) was approximately 1000 cells/mm^2^ (64.5% decrease from donor ECD) at 6-months postoperatively.

## 2 Discussion

In this case report, we demonstrated the usefulness of intraoperative OCT in nanothin DSAEK for a patient with a remarkably thickened cornea due to advanced bullous keratopathy. Previously, we demonstrated that visualization of intraocular structures including the retina was fair during DSAEK in 6 cases with usual bullous keratoplasty with a posterior segment problem.^14^ However, in the current patient with a rare case of an extremely thickened cornea, visibility of the anterior chamber was poor with a standard surgical microscope. With help of OCT images from longer wave-length infrared light, safe and appropriate maneuvers such as graft unfolding, air tamponade, and graft attachment were facilitated. Previously, Pasricha et al. reported successful intraoperative OCT-assisted DSAEK in 2 patients with nearly opaque corneas.^15^ They used a customized microscope-integrated OCT developed at Duke University.^15^ Intraoperative OCT can be also be helpful in long-standing stromal edema in congenital hereditary endothelial dystrophy.

Microscope-integrated OCT using the high-tech device will change the indication for corneal transplant procedures. Without an intraoperative OCT, surgeons might choose PK rather than DSAEK in patients with an extremely thickened cornea. It is well known that PK has a long-term risk of traumatic globe rupture.^16^ This devastating complication can be eliminated if patients undergo endothelial keratoplasty instead.

Preoperative evaluation with anterior segment OCT is also helpful for choosing DSAEK in patients with an extremely thickened cornea. In the current case, anterior segment OCT was able to exclude risk factors, such as significant irregularity of the posterior cornea, anterior iris synechiae, large iris defects, or an unstable intraocular lens.

In our patient, significant deswelling of the cornea, visual improvement, and pain relief were noted without any complications. In addition, postoperative ECD was fair (approximately 1000 cells/mm^2^ at 6 months). In a complex case in a patient with vision-limiting comorbidities, achieving functional vision by minimizing complications with DSAEK may be more important than achieving maximum vision potential with DMEK. Dunker et al. reported that graft detachment rate was higher in DMEK (24%) than in ultrathin DSAEK (4%).^17^ Delivering a nanothin graft with an NS endoinserter was relatively simple and easy, and the endothelial side of the donor tissue was ideally protected from compression.^18^ It should also be noted that a nanothin (≤50 μm thick) graft provides faster visual recovery than thicker DSAEK.^6^ However, we acknowledge that a thicker graft could also provide an excellent outcome in the current case. It is unclear whether the use of a nanothin graft enhances attachment for a host with posterior irregular stroma in comparison to a thicker graft.

The biggest limitation of intraoperative OCT is the high cost. And currently, few centers can use it in DSAEK.

In conclusion, intraoperative OCT is useful for nanothin DSAEK even when the surgical microscope view is compromised by an extremely thickened host cornea due to advanced bullous keratopathy. As an alternative to PK, less invasive nanothin DSAEK was successfully performed. Patients can benefit from the advantages of DSAEK over PK, such as fast vision recovery, and a low risk of traumatic globe rupture or suture complications.

## Patient consent

Written consent to publish this case has not been obtained. This report does not contain any personal identifying information.

## Acknowledgements and disclosures funding

No funding or grant support.

## Authorship

All authors attest that they meet the current ICMJE criteria for Authorship.

## Declaration of competing interest

All authors have no funding or conflicts of interest to disclose.
